# Glucagon‐Like Peptide‐1 Receptor Agonists and Anesthesia—Are We Clearer on the Correct Approach?

**DOI:** 10.1111/1753-0407.70041

**Published:** 2024-12-19

**Authors:** Zachary Bloomgarden

**Affiliations:** ^1^ Department of Medicine, Division of Endocrinology, Diabetes and Bone Disease Icahn School of Medicine Mount Sinai New York USA

A bit over a year ago, we argued in these pages against a consensus statement recommendation from the American Society of Anesthesiologists that patients taking glucagon‐like peptide‐1 receptor agonists (GLP‐1RA) should not take these agents for 1 week before elective procedures [1]. We pointed out the number of medications potentially interfering with gastric motility, including opiate analgesics, anticholinergics, antidepressants, calcium channel blockers, and gastric acid suppressants, with the lack of specific requirements that such treatments be held in preparation for anesthesia and sedation [1]. Some institutions now require that GLP‐1RA be withheld for several weeks before procedures, and indeed evidence based on drug levels suggests that “complete dissipation of the effect” would require 4–5 half‐lives, although this would likely be “potentially harmful” by virtue of requiring complete reorganization of diabetes treatment for many patients [2].

The imposition of these guidelines on people with diabetes has burdened them with the dilemma of not using important glucose‐lowering and appetite‐suppressing treatments with evidence of cardiovascular, renal, hepatic, and pulmonary benefits. Has there been interim progress?

In a study of 133 patients with type 2 diabetes undergoing 192 upper endoscopies, gastric contents were present in 19% of those taking versus 5% of those not taking a GLP‐1RA [3]. Another study of 124 patients with type 2 diabetes undergoing endoscopy found residual gastric contents in 56% versus 19% of those taking versus not taking a GLP‐1RA [4]. A study comparing 24 824 GLP‐1RA users with 18 541 sodium‐glucose cotransporter‐2 inhibitor users undergoing upper endoscopy, however, found similar pulmonary aspiration risks of 4.2 versus 4.3 per 1000, respectively, although endoscopy discontinuation rates were 9.8 versus 4.9 per 1000, respectively, the significantly greater risk with GLP‐1RA seen only among those with BMI ≥ 30 kg/m^2^, which the authors speculated might be related to retained gastric contents [5]. A study of 274 211 outpatient upper endoscopy procedures among individuals aged 18–64 with type 2 diabetes from 2005 to 2021 compared claims for aspiration and associated pulmonary adverse events in the 14 days following upper endoscopy, highlighting the infrequency of such events, with aspiration, aspiration pneumonia, and respiratory failure occurring with frequencies of 6.8, 7.6, and 25.6 cases per 10 000 endoscopies, respectively; there was no significant difference in event rates between users of GLP1‐RA and those of dipeptidyl peptidase 4 inhibitors (DPP4i), and significantly lower rates of pneumonia, respiratory failure, and hospitalization and ER visits among those using GLP‐1RA compared with individuals using chronic opioids, albeit without significant changes in documented aspiration or aspiration pneumonia [6]. Another study, however, did find a 1.33‐fold increase in the risk of aspiration pneumonia following endoscopy in GLP‐1RA users compared with nonusers, using propensity score matching to adjust for baseline differences; in total, 126 events occurred in 15 114 endoscopies among GLP‐1RA users, a rate of 8.3 per 1000 endoscopies [7].

A study of the occurrence of postoperative respiratory failure or aspiration pneumonia among patients with type 2 diabetes undergoing emergency surgery from 2015 to 2021 found identical adjusted event rates of 2.7% among the 3502 using versus 20 117 not using GLP‐1RA [8]. In a study of adults with diabetes undergoing surgery, 2256 of whom received a GLP‐1RA versus 11 405 receiving an oral hypoglycemic agent, postoperative clinical evidence of decelerated gastric emptying was seen in 21.5% versus 22.9%, aspiration pneumonia occurred in 0.4% versus 0.6%, respectively, and propensity score matching suggested a 19% lower rate of antiemetic use in those not using GLP‐1RA but no significant difference in 7‐day postoperative ileus, and no significant difference in postoperative aspiration pneumonia [9]. In a meta‐analysis of 14 studies of patients with type 2 diabetes undergoing surgery, GLP‐1RA use versus non‐use was associated with 9% versus 3% pre‐ and periprocedural gastrointestinal symptoms, without a significant increase in postoperative nausea or emesis, and with 8% versus 3% retained gastric contents, respectively; those receiving GLP‐1RA had improved glycemic control with a 61% lower requirement for postoperative insulin administration [10]. A minireview/literature search of studies involving GLP‐1RA therapy and adverse gastrointestinal/biliary events confirmed an association with retained gastric contents at the time of upper endoscopy, suggesting this to be related to delayed gastric emptying but noted, first, that longstanding type 2 diabetes accompanied by complications is strongly associated with delayed gastric emptying leading to retained gastric contents, and second, that this is infrequently associated with aspiration [11]. Thus, case reports of periprocedural pulmonary aspiration in patients with diabetes with (or without) GLP‐1RA treatment may simply be due to abnormality of gastric motility associated with long‐standing diabetes [12].

These considerations suggest little harm occurs with preprocedure use of GLP‐1RA, and have led to the US FDA recommending a change in recommendations for periprocedural GLP‐1RA use on November 1, 2024 [13, 14], stating, for semaglutide (with identical recommendations for liraglutide and tirzepatide), “There have been rare postmarketing reports of pulmonary aspiration in patients receiving GLP‐1 receptor agonists undergoing elective surgeries or procedures requiring general anesthesia or deep sedation who had residual gastric contents despite reported adherence to preoperative fasting recommendations. Available data are insufficient to inform recommendations to mitigate the risk of pulmonary aspiration during general anesthesia or deep sedation … including whether modifying preoperative fasting recommendations or temporarily discontinuing OZEMPIC could reduce the incidence of retained gastric contents” [15]. Guidance from the American Gastroenterological Association, American Society for Metabolic and Bariatric Surgery, American Society of Anesthesiologists, International Society of Perioperative Care of Patients with Obesity, and the Society of American Gastrointestinal and Endoscopic Surgeons gives three recommendations for periprocedural use of GLP‐1RA [16, 17]. First, ascertainment of the risk of delayed gastric emptying is suggested based on “gastrointestinal symptoms suggesting delayed gastric emptying; recent dose increases, higher doses, and weekly administered medications.” Unfortunately, there is little or no clinical trial or observational evidence that any of these reasonable‐seeming risk factors are actually related to outcome. “Medical conditions beyond GLP‐1RA usage, which may also delay gastric emptying,” are additional suggested risks. The second recommendation states, “Continue GLP‐1RA therapy preoperatively if there is no concern for delayed gastric emptying.” However, diabetes is such a medical condition, that this recommendation continues to suggest “liquid only diet for at least 24 h before procedure with usual recommended fasting protocol, or … medication bridging if GLP‐1RAs need to be discontinued.” Following this is the third recommendation, to “proceed with procedure if there is no concern for delayed gastric emptying … [otherwise] consider point‐of‐care gastric ultrasound and/or … rapid sequency induction of general anesthesia.” The authors stress that this “should be considered guidance and not an evidence‐based guideline,” leaving us with questions but no real answers.

How are we to proceed? An analysis in The Medical Letter interprets the new recommendations to “suggest that most patients can continue taking these drugs during the perioperative period” [18]. This may well be the best approach. Endoscopic studies do document a higher likelihood of retained gastric content during endoscopy in people with type 2 diabetes receiving a GLP‐1RA, but two of the three large clinical dataset analyses showed no increase in aspiration pneumonia [5, 6] and in the third, there was a 33% increase in the risk of events, occurring in 0.83% of those receiving a GLP‐1RA and in 0.63% of those not receiving these agents [7]. Two studies of patients with type 2 diabetes undergoing surgery failed to show an increase in aspiration or other complications [8, 9]. Meta‐analyses support these conclusions. Given the many benefits of GLP‐1RA treatment in people with diabetes (as well as in those with obesity and heart failure or renal disease), a reasonable approach in the light of the new FDA and society guidance may be to continue these medications, and in patients with symptoms of gastroparesis to use a liquid diet preoperative approach with fasting diabetes treatment adjustment to further reduce any chance of aspiration.

## Conflicts of Interest

The author declares no conflicts of interest.
